# Analysis of influencing factors of washed microbiota transplantation in treating patients with metabolic syndrome

**DOI:** 10.3389/fnut.2025.1508381

**Published:** 2025-02-03

**Authors:** De-Jiang Lin, Dong-Xia Hu, Qing-Ting Wu, Lin-Gui Huang, Zi-Han Lin, Jia-Ting Xu, Xing-Xiang He, Lei Wu

**Affiliations:** ^1^Department of Gastroenterology, Research Center for Engineering Techniques of Microbiota-Targeted Therapies of Guangdong Province, The First Affiliated Hospital of Guangdong Pharmaceutical University, Guangzhou, China; ^2^School of Biological Sciences and Engineering, South China University of Technology, Guangzhou, China

**Keywords:** fecal microbiota transplantation, washed microbiota transplantation, metabolic syndrome, logistic regression, influencing factors

## Abstract

**Background and aims:**

Metabolic Syndrome (MS) is a cluster of metabolic abnormalities closely associated with hypertension, diabetes, hyperlipidemia, obesity, etc. Our previous research indicated that fecal microbiota transplantation (FMT) could improve MS, but the factors influencing the efficacy of washed microbiota transplantation (WMT) in treating MS patients remain unclear. The objective of this study is to analyze the influencing factors of WMT in treating MS patients.

**Methods:**

The clinical data and influencing factors related to MS patients were collected retrospectively. Not only the changes in body mass index [BMI = weight (kg)/height (m)^2^], blood glucose, blood lipids, and blood pressure were analyzed, but also the influencing factors of WMT in treating MS patients were carried out based on Logistic Regression. The 16S rRNA gene amplicon sequencing was performed on fecal samples before and after WMT treatment.

**Results:**

A total of 210 patients were included, including 68 patients in the WMT group and 142 patients in the drug treatment (DT) group. WMT had a significant improvement and ASCVD downregulation effect on MS patients, and 42.65% of MS patients removed the label of MS after WMT treatment. Independent influencing factors for treating MS patients through WMT include age < 60 years old, high smoking index, infection, single donor selection, single-course WMT treatment, and having hypertension, diabetes, or obesity. WMT treated MS patients by maintaining the balance of gut microbiota.

**Conclusions:**

WMT has a significant effect in improving MS and downregulating ASCVD risk stratification. The therapeutic effect of WMT on MS patients is closely related to their age, smoking index, infection, chronic disease status, donor type, and WMT courses. Therefore, we can improve the efficacy of WMT by reducing independent influencing factors that affect gut microbiota homeostasis.

## 1 Introduction

Metabolic Syndrome (MS) is a group of clinical syndromes combined with obesity, hyperglycemia (diabetes or impaired glucose regulation), dyslipidemia (hyperglycemia and/or low HDL-c hyperemia) and hypertension, which increases the risk of type 2 diabetes mellitus by fivefold and cardiovascular disease by threefold (1). In addition, MS is closely associated with non-alcoholic fatty liver disease (NAFLD), chronic kidney disease, polycystic ovary syndrome, cancers such as liver cancer, colorectal cancer and kidney cancer (2–5). The two main potential risk factors of MS are obesity and insulin resistance, while aggravating factors include physical inactivity, aging, endocrine and genetic factors, characterized by high incidence rate, diverse clinical manifestations, complex mechanisms, and difficult treatment (1, 2). The latest data shows the prevalence of MS in the population aged 20 and above is as high as 31.1% in China, and the global prevalence is still rising, becoming a major global health hazard (6, 7).

Beyond changing lifestyle and dietary structure, the current main methods for treating MS include strengthening aerobic exercise and drug treatment (8). Unlike traditional drugs, fecal microbiota transplantation (FMT) is a new treatment method utilizing healthy microbiota to replace imbalanced microbiota in patients, thereby restoring normal gut microbiota function and maintaining various neurological and metabolic functions (9–11). Numerous studies had confirmed the close correlation between gut microbiota and MS, suggesting that improving gut microbiota could reduce insulin resistance, enhance fat utilization, promote the absorption of blood pressure regulating substances and restore the microbial biological environment (12–14). Washed microbiota transplantation (WMT) is a microbial community transplantation method similar to FMT, which increases the process of washing and filtering microbial communities. Compared with FMT, the bacterial solution of WMT is prepared by the intelligent microbial isolation system (GenFMTer), which is filtered through a multi-level filtration system to screen for adverse inflammatory factors that cause human inflammation, resulting in a safer and more effective bacterial solution (15).

Our previous research had shown that WMT had a significant improvement effect on MS patients by restoring their gut microbiota homeostasis (16), laying the foundation for the clinical application of FMT to treat MS patients. In addition, our study also confirmed that WMT did not further increase the blood lipids, blood glucose, and blood pressure of non-metabolic syndrome patients, thereby reducing the interference factors in the study (16). However, there were a number of MS patients who could not remove the label of MS after WMT treatment, and the factors that affect the efficacy of WMT in treating MS patients were still unknown. To treat MS patients effectively, we tried to explore the influencing factors of WMT in treating MS patients in the Department of Gastroenterology at the First Affiliated Hospital of Guangdong Pharmaceutical University. Therefore, we conducted a retrospective trial to collect clinical data of MS patients receiving WMT or drug treatment.

## 2 Materials and methods

### 2.1 Patients and experimental design

This study included MS patients who completed 1–4 courses of WMT treatment or ordinary treatment in the First Affiliated Hospital of Guangdong Pharmaceutical University from January 2017 to December 2023 during the first WMT. Inclusion criteria were patients aged 18–80 with informed consent and voluntary acceptance of WMT. Exclusion criteria were pregnant women and patients who changed their hypoglycemic, antihypertensive, or lipid-lowering medications during the observation period. The study was approved by the Ethics Committee of the First Affiliated Hospital of Guangdong Pharmaceutical University, adhering to the Helsinki Declaration (No.2021-13). Among the enrolled participants, 210 met the requirements of this study and provided written informed consent to participate.

The diagnostic criteria for MS in this study refer to the Chinese Guidelines for the Prevention and Treatment of Type 2 Diabetes (2020 Edition) (17) and the diagnostic criteria for metabolic syndrome of the Diabetes Society of the Chinese Medical Association (18). Metabolic syndrome was diagnosed with three or more of the following: (1) BMI ≥ 25 kg/m^2^. (2) Hyperglycemia: fasting blood glucose ≥6.1 mmol/L or blood glucose ≥7.8 mmol/L 2 h after glucose load and (or) those who have been diagnosed with diabetes and received treatment. (3) Hypertension: Blood pressure ≥ 130/85 mmHg (1 mmHg = 0.133 kPa) and (or) diagnosed with hypertension and receiving treatment. (4) Fasting triglycerides (TG) ≥ 1.70 mmol/L. (5) Fasting HDL-c < 1.04 mmol/L. The 210 patients included in this study were divided into WMT group and DT group based on whether they received WMT treatment. There were a total of 68 patients in the WMT group, and 142 patients in the DT group who were hospitalized at the same time but did not receive WMT treatment or take medication such as microbial preparations and probiotics.

In accordance with the Chinese Guidelines for the Prevention of Cardiovascular Diseases (2017 Edition) (19), ASCVD risk stratification was carried out according to the baseline and blood lipid status, which were classified extremely high risk group, high risk group, medium risk group, and low risk group. Subsequently, patients were divided into the single-course WMT group, double-course WMT group, and multi-course WMT group (three or more courses) for WMT. After receiving 1–4 rounds of WMT or drug treatment and completing follow-up, statistical analysis and evaluation of height, weight, blood glucose, blood lipids, and blood pressure results were conducted for all patients.

### 2.2 Preparation and treatment of washed microbiota transplantation

The WMT program adhered to the Nanjing Consensus on the Methodology of Washed Microbial Transplantation (20). All healthy donors aged between 18 and 25 must undergo rigorous counseling, psychological and physical examinations, biochemical tests, and screening for infectious diseases. The specific fecal preparation procedures can refer to the Nanjing Consensus (20) and our previous research. Relevant indicators were collected before completing each course of treatment, including baseline values, values after the first course (single-course), values after the second course (double-course), and values after the third or more courses (multi-course).

### 2.3 Clinical and follow-up data collection

The study collected data from MS patients before (baseline) and after treatment, and compared the partial clinical efficacy between the WMT group and the DT group using the difference before and after treatment as the improvement value. Mainly including BMI indicators: weight (kg), height (m), weight/height^2^ = BMI (kg/m^2^). Blood glucose indicators: fasting blood glucose (FBG, mmol/L), fasting insulin (FI, mU/mL), and the insulin resistance value (HOMA-IR, insulin resistance value = fasting blood glucose ^*^ fasting insulin/22.5). Blood lipid indexes: total cholesterol (TC, mmol/L), triglyceride (TG, mmol/L), low density lipoprotein cholesterol (LDL-c, mmol/L), high density lipoprotein cholesterol (HDL-c, mmol/L). Blood pressure indicators: systolic blood pressure (SBP, mmHg) at admission, and diastolic blood pressure (DBP, mmHg) at admission. And various influencing factors: age, gender, smoking and alcohol history, medication and infection status, chronic disease, pathways of WMT, selection of donor type and number of WMT courses. Smoking index = number of cigarettes smoked/day × Years of smoking (smoking index < 200 represents mild smoking, while smoking index 200 ≥ moderate to severe smoking). The middle digestive tract is defined as microbiota transplantation through a jejunal tube, while the lower digestive tract is microbiota transplantation through a colon tube. Adverse events (AEs): diarrhea, fever, fatigue, nausea, abdominal pain, etc.

### 2.4 DNA extraction and sequencing

The fecal samples from 5 MS patients who excluded independent influencing factors and 5 donors were collected before and after WMT for sequencing. After collection, all samples were stored at −80°C until DNA was extracted. DNA quality and concentration were checked by NanoDrop™ 2000 (Thermo Fisher Scientific, Wilmington, DE, USA) (21). Primers 338F (50-ACTCCTACGGGAGGCAGCAG-30) and 806R (50-GGACTACHVGGGTWTCTAAT-30) were used to amplify bacterial 16S rRNA gene fragments (V3-V4) from extracted DNA. The PCR products were subjected to agarose gel electrophoresis to determine the size of the amplicon. The constructed library was quantified by Qubit and Q-PCR, and the NovaSeq6000 (Illumina, San Diego, CA, USA) sequencing platform was used for onboard sequencing until the library was qualified (22).

### 2.5 Amplicon data processing and analysis

From all the sample data split from plane data and amputation of barcode and primer sequences after the use of FLASH software (version 1.2.11, http://ccb.jhu.edu/software/FLASH/) to splice the sample reads, raw tags were obtained (23). Then, fastp software version 0.23.1 (Shenzhen Hypros, Shenzen, China) was used to obtain high-quality clean tags (24). Finally, clean tags were compared with the database to detect and remove chimeras, so as to obtain the effective tags (25). The DADA2 Variants in QIIME2 were used to obtain the final ASV variants and the feature list of the variant. The resulting ASVs were then compared with the database by the classify-sklearn module in QIIME2 software version 2.0 (QIIME 2 development team, https://docs.qiime2.org) to obtain species information for each ASV (26). The representative sequences of ASVs using the classification sklearn (Naive Bayes) algorithm were analyzed, obtaining the relative abundance of ASVs at the genus level.

### 2.6 Statistical analysis

Statistical analysis was performed using SPSS 22.0 (IBM Corp, Armonk, NY, USA) and Prism 8 (GraphPad, San Diego, CA, USA). The Categorical variables were analyzed by Chi-squared test or Fisher exact test. For comparison of continuous variables between two independent groups, unpaired Student's *t-*test (Normal distribution) and Mann Whitney test (non-Normal distribution) could be used. The paired data were compared by paired Student's *t-*test (Normal distribution) and Wilcoxon signed rank test (non-Normal distribution). Logistic regression analysis is used to summarize the main factors that affect therapeutic efficacy. Two-tailed *p-*values < 0.05 were considered statistically significant.

## 3 Results

### 3.1 Clinical characteristics of patients in WMT and DT group

A total of 210 patients met the inclusion criteria for WMT treatment or ordinary treatment at the First Affiliated Hospital of Guangdong Pharmaceutical University from January 2017 to December 2023, including 126 males (60.00%) and 84 females (40.00%), with an average age of 60.12 ± 11.66 years. Due to different patient compliance, each WMT treatment might not be completed as scheduled. The average number of microbiota transplants is 2.68 ± 1.22, which is close to 3 times. This study calculated the time interval of selected patients, expressed in days as the median (25%−75%). The blood test results of the patient before the first treatment were the baseline values, with a treatment interval of 36 days (32–46 days) in single-course WMT, 95 days (76.75–104.25 days) in double-course WMT, 192 days (152.75–212.50 days) in multi-course WMT, and 42 days (36.42–50.48 days) in the drug treatment (DT) group.

The comparison of demography and clinical characteristics between the WMT and the DT group is shown in Table 1. Due to different compliance, not all patients had complete data, so the number of patients in each group was different for each indicator. There was no significant difference in age, gender, medical history, and laboratory indicators between the WMT group and DT group in MS patients, which reduces the interfering factors for our study of efficacy differences between treatment groups. Compared to MS patients, the various indicators of the donor, including BMI, blood glucose, blood lipid, and blood pressure, were healthier.

**Table 1:** Demographics and clinical characteristics of patients and donors at baseline.

	**WMT group (*n =* 68)**	**DT group (*n =* 142)**	***p-*value**	**Donors (*n =* 8)**
Age (year)	58.35 ± 13.53 (*n =* 68)	60.97 ± 10.60 (*n =* 142)	0.128	22.80 ± 0.84 (*n =* 8)
Hypertensive patients *n* (%)	41 (60.29)	85 (59.86)	0.976	5 (62.50)
Type 2 diabetes patients *n* (%)	32 (47.06)	108 (76.06)	0.053	/
Male *n* (%)	39 (57.35)	109 (76.76)	0.220	/
BMI (kg/m^2^)	26.61 ± 4.67 (*n =* 68)	26.35 ± 3.65 (*n =* 142)	0.664	21.26 ± 1.23 (*n =* 8)
FBG (mmol/L)	6.23 ± 2.34 (*n =* 68)	6.72 ± 2.27 (*n =* 142)	0.152	4.60 ± 0.24 (*n =* 8)
HbA1c (%)	6.94 ± 1.31 (*n =* 29)	7.41 ± 1.66 (*n =* 131)	0.164	/
FI (mU/mL)	11.78 (8.40–14.78) (*n =* 35)	10.72 (6.30–19.54) (*n =* 24)	0.678	/
HOMA-IR	3.16 (2.58–4.58) (*n =* 35)	3.16 (2.58–4.58) (*n =* 24)	0.568	/
TC (mmol/L)	4.67 (3.92–5.76) (*n =* 68)	4.51 (3.81–5.26) (*n =* 139)	0.189	4.34 ± 0.59 (*n =* 8)
TG (mmol/L)	1.97 (1.28–3.30) (*n =* 68)	2.02 (1.59–2.61) (*n =* 139)	0.742	0.79 ± 0.36 (*n =* 8)
LDL-c (mmol/L)	2.65 ± 1.03(*n =* 68)	2.54 ± 0.95 (*n =* 139)	0.478	2.32 ± 1.21 (*n =* 8)
HDL-c (mmol/L)	0.96 (0.84–1.17) (*n =* 68)	0.95 (0.85–1.14) (*n =* 139)	0.986	1.25 ± 0.36 (*n =* 8)
SBP (mmHg)	133.12 ± 12.01 (*n =* 68)	136.12 ± 18.04 (*n =* 142)	0.126	122.40 ± 10.64 (*n =* 8)
DBP (mmHg)	78.56 ± 11.13 (*n =* 68)	80.78 ± 9.69 (*n =* 142)	0.214	74.00 ± 7.97 (*n =* 8)

Data is represented as mean ± standard deviation, interquartile spacing or n (%). BMI (kg/m^2^), Body mass index; FBG (mmol/L), Fasting blood glucose; HbA1c (%), Glycated hemoglobin; FI (μU/mL), Fasting insulin; HOMA-IR, Homeostasis model assessment of insulin resistance; TC (mmol/L), Total cholesterol; TG (mmol/L), Triglyceride; LDL-c (mmol/L), Low-density lipoprotein cholesterol; HDL-c (mmol/L), High-density lipoprotein cholesterol; SBP (mmHg), Systolic blood pressure; DBP (mmHg), Diastolic blood pressure.

### 3.2 Comparative analysis of each index after and baseline in the WMT group

**Figure 1:**
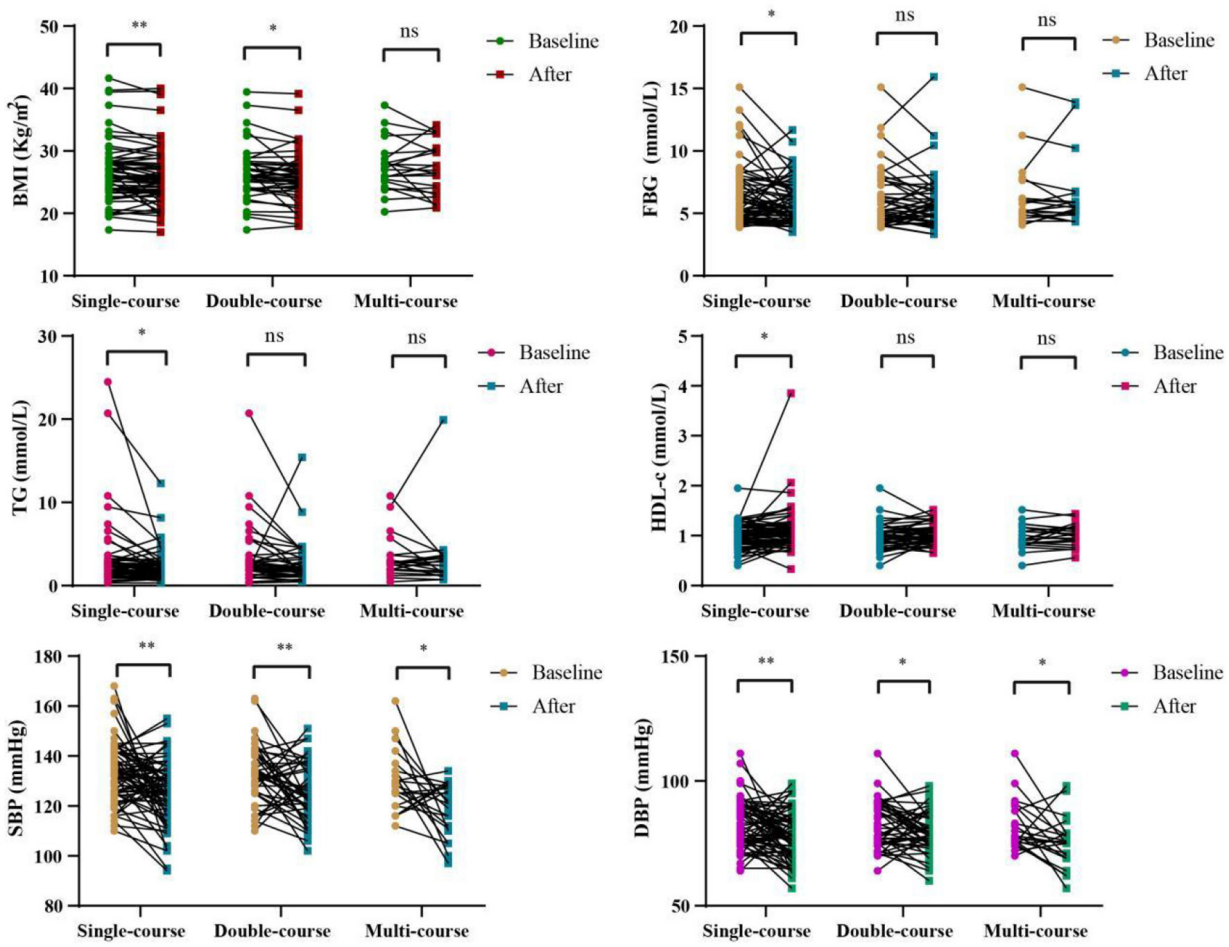
Changes of BMI, FBG, TG, HDL-c, SBP, DBP in different courses of in the WMT group; *indicates *p* < 0.05, **indicates *p* < 0.01; ns, not significant. All data can be found on Supplementary Table S1.

Figure 1 and Supplementary Table S1 show the impact of WMT on BMI, blood glucose, blood lipids and blood pressure in MS patients in the WMT group. The results showed that single-course treatment had a significant reducing effect on BMI (from 26.61 ± 4.67 to 26.05 ± 4.61, *p* = 0.007), FBG (from 6.36 ± 2.37 to 5.83 ± 1.76, *p* = 0.038), TG (from 3.20 ± 4.04 to 2.34 ± 1.87, *p* = 0.013), HDL-c (from 0.99 ± 0.25 to 1.10 ± 0.45, *p* = 0.029), SBP (from 133.12 ± 12.01 to 125.78 ± 12.56, *p* < 0.001) and DBP (from 82.78 ± 9.69 to 77.81 ± 9.40, *p* = 0.002). At the same time, the double-course treatment also showed a significant reducing effect on BMI (from 26.44 ± 4.30 to 25.74 ± 4.28, *p* = 0.033) and SBP (from 132.49 ± 11.82 to 125.64 ± 11.64, *p* = 0.006), DBP (from 82.56 ± 9.65 to 79.18 ± 8.23, *p* = 0.037), while the multi-course treatment only had a significant reducing effect on SBP (from 129.82 ± 12.25 to 118.68 ± 10.51, *p* = 0.010) and DBP (from 82.05 ± 9.96 to 75.18 ± 10.05, *p* = 0.024).

### 3.3. Comparison of improvement values for various indicators in the WMT group

**Figure 2:**
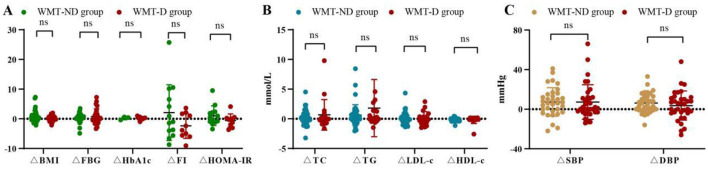
Comparison of improvement values for various indicators during the single-course of treatment in the WMT group. ΔBMI refers to the improvement value in BMI, and so on. WMT-ND group represents WMT without drug treatment and WMT-D group represents WMT plus drug treatment. **(A)** Comparison of blood glucose indicators and BMI improvement values between two groups. **(B)** Comparison of blood lipid indicators and BMI improvement values between two groups. **(C)** Comparison of blood pressure indicators and BMI improvement values between two groups; ns, not significant. All data can be found on Supplementary Table S2.

Figure 2 and Supplementary Table S2 show the comparison of improvement values for various indicators under different conditions during the single-course of treatment in the WMT group. Regarding blood glucose indicators and BMI in Figure 2A, the WMT plus drug (WMT-D) group showed superior effects to the WMT without drug (WMT-ND) group in reducing FBG, while the WMT-ND group was superior in reducing BMI, HbA1c, FI, and HOMA-IR. For blood lipid indicators in Figure 2B, the WMT-D group had a better improvement effect than the WMT-ND group on TC, TG, LDL-c, and HDL-c. However, there was not much difference between the two groups in terms of blood pressure reduction in Figure 2C. In our study, even though some indicators of the WMT-D group were superior to those of the WMT-ND group, there was no statistically significant difference between the two groups as a whole, indicating that WMT can improve indicators such as blood glucose, blood lipids and blood pressure in MS patients.

### 3.4 Comparison of clinical efficacy in WMT and DT group

**Figure 3:**
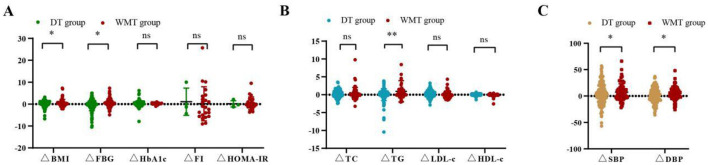
Comparison of improvement values for various indicators between the DT group and WMT group. **(A)** Comparison of blood glucose indicators and BMI improvement values between two groups. **(B)** Comparison of blood lipid indicators and BMI improvement values between two groups. **(C)** Comparison of blood pressure indicators and BMI improvement values between two groups; *indicates *p* < 0.05, **indicates *p* < 0.01; ns, not significant. All data can be found on Supplementary Table S3.

As shown in Figure 3 and Supplementary Table S3, compared to the DT group, the WMT group had greater advantages in reducing blood glucose, blood pressure, lipid, and weight, such as FBG improvement value (0.53 ± 1.99 vs. −0.41 ± 2.57, *p* = 0.012), TG improvement value (0.96 ± 2.97 vs. 0.02 ± 1.67, *p* = 0.006), SBP improvement value (7.34 ± 15.86 vs. 1.97 ± 18.00, *p* = 0.037), DBP improvement value (4.97 ± 12.52 vs. 0.99 ± 13.74, *p* = 0.045), and BMI improvement value (0.56 ± 1.64 vs. 0.12 ± 1.33, *p* = 0.047).

As shown in Table 2, WMT had the effect of treating MS patients as non-MS patients (*p* = 0.011), with 29 (42.65%) MS patients improving to non-MS patients in the WMT group. Therefore, WMT treatment was superior to drug therapy in treating MS patients effectively.

**Table 2:** Clinical comprehensive efficacy evaluation based on diagnostic level of MS and ASCVD risk stratification.

**Therapeutic effect base on diagnostic level of MS**						**Therapeutic effect base on ASCVD risk stratification**					
**Groups**	**Before therapy (** ***n*** **)**	**Unchanged group (** ***n*** **)**	**Changed group (** ***n*** **, %)** ^*^	*X* ^2^	***p-*** **value**	**Groups**	**Before therapy (** ***n*** **)**	**Unchanged group (** ***n*** **)**	**Changed group (** ***n*** **, %)** ^**^	*X* ^2^	***p-*** **value**
**High-risk group**											
WMT group	68	39	29 (42.65)	6.435	0.011	WMT group	31	17	14(45.16)	6.435	0.011
DT group	142	106	36 (25.35)			DT group	39	32	7 (17.95)		
**Medium-risk group**											
						WMT group	29	15	14 (48.28)	4.300	0.038
						DT group	24	19	5 (20.83)		
**Low-risk group**											
						WMT group	2	2	0 (0.00)	0.001	0.999
						DT group	4	3	1 (25.00)		

Data is represented as n or %.

^*^The definition of the unchanged group is still the MS group, while the changed group is the non-MS group.

^**^The unchanged group is defined as a grade decline in the high-risk and medium-risk group, while it is defined as a grade increase in the low-risk group.

In addition, according to ASCVD risk stratification (19), MS patients were divided into extremely high-risk group, high-risk group, medium-risk group and low-risk group. Acute coronary syndrome, stable coronary heart diseases, ischemic cardiomyopathy, peripheral atherosclerosis and ischemic stroke were included in the extremely high-risk group. This group of patients was not reassigned after WMT treatment and is not listed in Table 2.

In the high-risk group, 14 (45.16%) patients experienced a grade decline, which reduced high-risk ASCVD stratification from 45.59% to 25.00% in the WMT group (*p* = 0.011). In the medium-risk group, 14 (48.28%) patients experienced a grade decline, which reduced medium-risk ASCVD stratification from 42.65% to 22.06% (*p* = 0.038). In the low-risk group, this change was not statistically significant, indicating that WMT treatment did not increase the risk of ASCVD. In conclusion, WMT has a significant ASCVD downgrade effect on high-risk and medium-risk MS patients.

Among the completed WMT treatments, the total adverse reaction rate was 2.96%. Only one patient experienced fever with a maximum body temperature of 37.6°C, along with diarrhea after treatment completion. All symptoms were alleviated after < 3 days of symptomatic treatment.

### 3.5 Analysis of factors influencing the efficacy of WMT

This study included 135 MS patients in the WMT group, including 68 patients who completed a single course of WMT, 45 patients who completed a dual course of WMT, and 22 patients who completed multiple courses of WMT. Among the 135 MS patients, 55 patients recovered to non-MS patients after treatment, with a success rate of 40.74%.

It showed the influencing factors that may affect the efficacy of WMT in treating MS patients in Tables 3, 4, including antibiotics and immunosuppressants use, infection with pathogenic bacteria or viruses during observation, hypertension, diabetes, hyperlipemia, obesity, other chronic diseases, the pathways of WMT, the selection of donor type and number of WMT courses. As shown in Table 4, in both the MS group and non-MS group formed after WMT treatment, the MS group had a significantly higher proportion of age < 60 years old, smoking index ≥200, using of antibiotics or immunosuppressants during observation, infection with pathogenic bacteria or viruses during observation, having hypertension, diabetes, obesity, single donor selection and single-course WMT treatment compared to the non-MS group (*p* < 0.050), suggesting the main factors affecting the efficacy of WMT in treating MS patients. However, there was not currently statistical significance in gender, alcohol consumption, having hyperlipidemia, suffering from other chronic diseases and pathways of WMT.

**Table 3:** The assignment result of the independent variable in the WMT group.

**Independent variable**	**Assignment**	**Independent variable**	**Assignment**
Gender (X1)	Male = 0, female = 1	Having diabetes (X9)	Yes = 1, None = 0
Age (X2)	< 60 years old = 0, ≥60 years old = 1	Having hyperlipidemia (X10)	Yes = 1, None = 0
History of drinking (X3)	Drinking = 0, not drinking and abstaining from drinking = 1	Having Obesity (BMI ≥ 28kg/m^2^) (X11)	Yes = 1, None = 0
History of smoking (X4)	Smoking index ≥200 = 0, < 200 = 1	Suffering from other chronic diseases (X12)	Yes = 1, None = 0
Use of antibiotics during observation (X5)	Yes = 1, None = 0	Pathways of WMT (X13)	Middle gastrointestinal tract = 0, lower gastrointestinal tract = 1
Use of immunosuppressants during observation (X6)	Yes = 1, None = 0		
Infection with pathogenic bacteria or viruses (X7)	Yes = 1, None = 0	Selection of donor type (X14)	Single donOR = 0, multiple donors = 1
Having hypertension (X8)	Yes = 1, None = 0	Number of WMT courses (X15)	Single-course = 0, double and multiple course = 1

**Table 4:** Comparison of independent variable composition after WMT treatment.

**Possible influencing factors**		**MS group (*n =* 80)**	**Non-MS group (*n =* 55)**	***X* ^2^**	***p-*value**
Gender (X1)	Male	50 (62.50)	29 (52.73)	1.282	0.257
	Female	30 (37.50)	26 (47.27)		
Age (X2)	< 60 years old	45 (56.25)	21 (38.18)	4.258	0.039
	≥60 years old	35 (43.75)	34 (61.82)		
History of drinking (X3)	Drinking	10 (12.50)	5 (9.09)	0.384	0.536
	Not drinking and abstaining from drinking	70 (87.50)	50 (90.91)		
History of smoking (X4)	Smoking index ≥200	16 (20.00)	1 (1.82)	9.789	0.002
	Smoking index < 200	64 (80.00)	54 (98.18)		
Use of antibiotics during observation (X5)	Yes	15 (17.65)	1 (1.82)	8.944	0.003
	None	65 (82.35)	54 (98.18)		
Use of immunosuppressants during observation (X6)	Yes	16 (18.82)	2 (3.64)	7.552	0.006
	None	64 (81.18)	53 (96.36)		
Infection with pathogenic bacteria or viruses (X7)	Yes	25 (29.41)	2 (3.63)	15.533	< 0.001
	None	62 (70.59)	53 (96.37)		
Having hypertension (X8)	Yes	53 (66.25)	25 (45.45)	5.778	0.016
	None	27 (33.75)	30 (54.55)		
Having diabetes (X9)	Yes	46 (57.50)	16 (29.09)	10.592	0.001
	None	34 (42.50)	39 (70.91)		
Having hyperlipidemia (X10)	Yes	41 (51.25)	23 (41.82)	1.163	0.281
	None	39 (48.75)	32 (58.18)		
Having obesity (BMI ≥28kg/m^2^) (X11)	Yes	36 (45.00)	12 (21.82)	11.416	0.001
	None	34 (55.00)	33 (78.18)		
Suffering from other chronic diseases (X12)	Yes	22 (27.50)	14 (25.45)	0.070	0.792
	None	58 (72.50)	41 (74.55)		
Method of WMT (X13)	Middle gastrointestinal tract	37 (46.21)	21 (38.18)	0.866	0.352
	Lower gastrointestinal	43 (53.79)	34 (61.82)		
Selection of donor type (X14)	Single donor	20 (25.00)	27 (49.09)	8.335	0.004
	Multiple donors	60 (75.00)	28 (50.91)		
Number of WMT treatment sessions (X15)	Single-course	48 (60.00)	20 (36.36)	7.284	0.007
	Double and multiple courses	32 (40.00)	35 (63.64)		

Data is represented as n or %; *p* < 0.05 were considered statistically significant.

**Figure 4:**
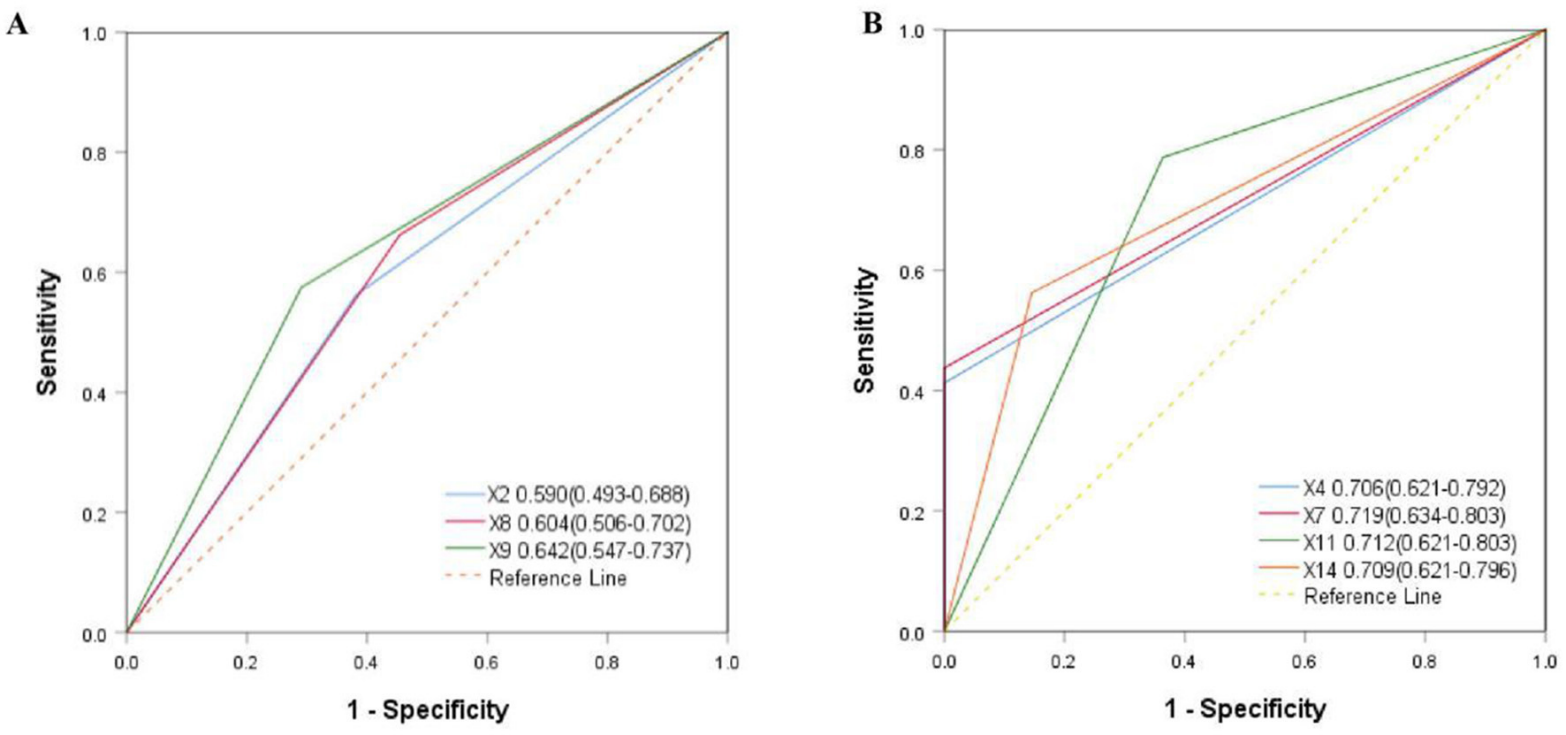
Comparison of area under AUC aurve for independent influencing factors. **(A)** X4 = smoking index, X7 = infection with bacteria or viruses during observation, X11 = having obesity, X14 = selection of donor type. **(B)** X2 = age, X8 = having hypertension, X9 = having diabetes.

Based on the logistic regression analysis in Table 5, not only the age < 60 years old (OR = 3.525, *p* = 0.023), smoking index ≥200 (OR = 11.705, *p* = 0.034), infection of pathogenic bacteria or viruses during observation (OR = 8.783, *p* = 0.037), single donor selection (OR = 3.003, *p* = 0.026) and single-course WMT (OR = 2.721, *p* = 0.041), but also suffering from hypertension (OR = 3.375, *p* = 0.026), diabetes (OR = 3.928, *p* = 0.008), and obesity (OR = 2.945, *p* = 0.038) were independent influencing factors. As shown in Figure 4A, the area under the AUC curve of some independent influencing factors was >0.700, including smoking index (X4), area under AUC curve = 0.706 (0.621–0.792), infection with pathogenic bacteria or viruses during observation (X7), area under AUC curve = 0.719 (0.634–0.803), having obesity (X11), area under AUC curve = 0.712 (0.621–0.803), selection of donor type (X14), and area under AUC curve = 0.712 (0.624–0.796), indicating that the model had a certain degree of predictability. However, some independent influencing factors had an area under the AUC curve < 0.700 in Figure 4B.

**Table 5:** Logistic regression analysis of the influencing factors of WMT on the efficacy of MS patients.

**Influencing factors**	**β**	**SE**	**Wald**	***p-*value**	**OR**	**95%CI**
Age < 60 years old vs. ≥60 years old (X2)	1.260	0.554	5.179	0.023	3.525	1.191–10.435
Smoking index ≥200 vs. < 200 (X4)	2.460	1.158	4.512	0.034	11.705	1.209–113.292
Use of antibiotics during observation yes vs. none (X5)	0.311	1.367	0.052	0.820	1.365	0.094–19.871
Use of immunosuppressants during observation yes vs. none (X6)	0.673	0.902	0.556	0.456	1.959	0.334–11.488
Infection with pathogenic bacteria or viruses yes vs. none (X7)	2.173	1.041	4.356	0.037	8.783	1.141–67.585
Having hypertension yes vs. none (X8)	1.217	0.545	4.984	0.026	3.375	1.160–9.822
Having diabetes yes vs. none (X9)	1.368	0.517	7.003	0.008	3.928	1.426–10.822
Having obesity yes vs. none (X11)	1.080	0.522	4.284	0.038	2.945	1.059–8.187
Selection of single donor vs. multiple donors (X14)	1.100	0.493	4.968	0.026	3.003	1.142–7.897
Single-course vs. double and multiple courses (X15)	1.001	0.489	4.190	0.041	2.721	1.043–7.097

### 3.6 Analysis of gut microbiota composition before and after WMT

**Figure 5:**
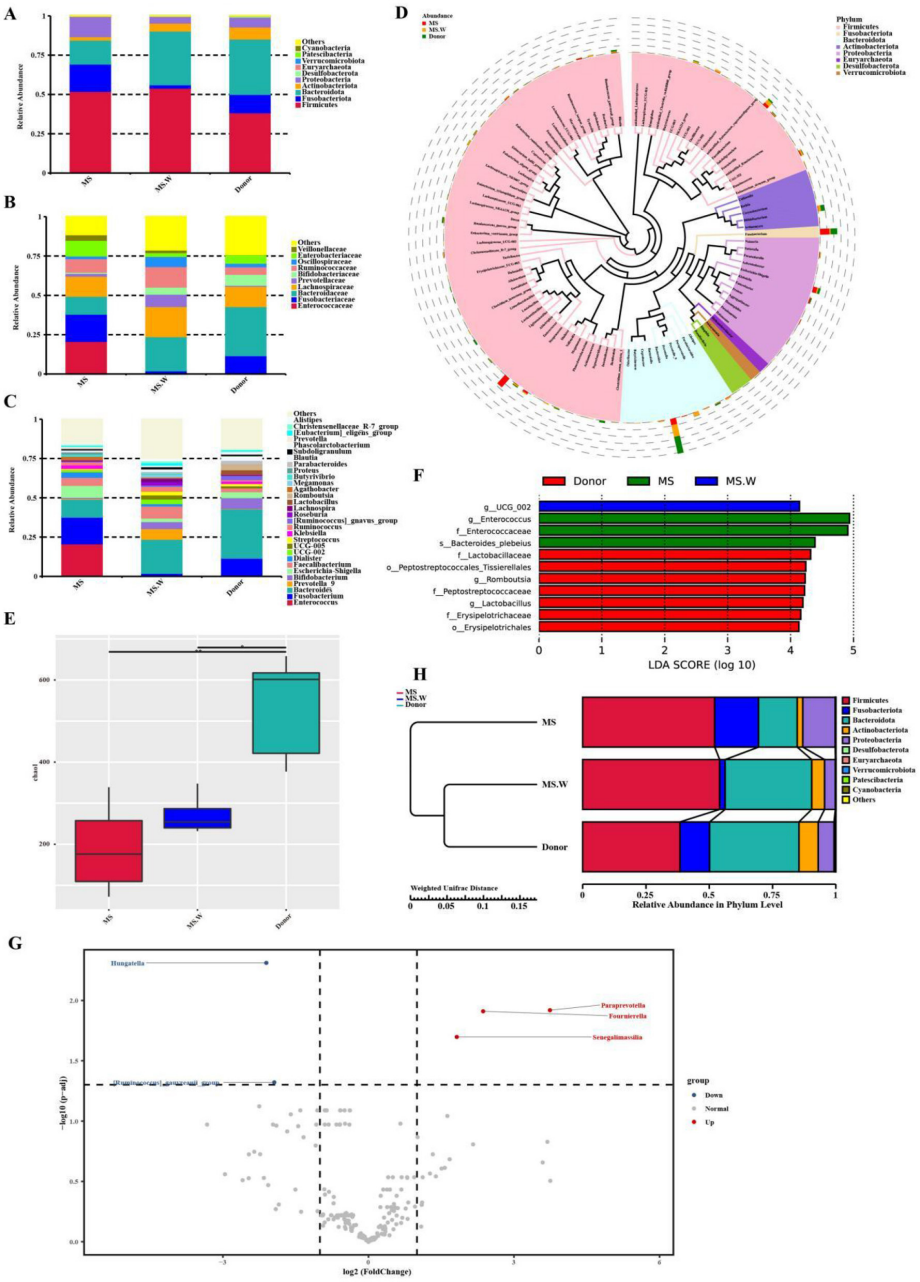
The composition of gut microbiota before and after WMT. **(A)** The composition of the top ten phyla of gut microbiota. **(B)** The composition of gut microbiota in the top ten families. **(C)** The composition of the gut microbiota of the top 30 genera. **(D)** The phylogenetic relationships of the top 100 genera of gut microbiota. **(E)** Chao1 index α diversity analysis. **(F)** LEfSe analysis of the MS patients and donors. **(G)** Analysis of metastatic lesions before and after WMT in MS patients. **(H)** Comparison of the abundance of the top five microbiota at the phylum level in MS patients before and after WMT, as well as in donors. MS: in the MS group before WMT. MS.W: in the MS group after WMT. d1-5: the first donor to the fifth donor. ^*^indicates *p* < 0.05, ^**^indicates *p* < 0.01.

We analyzed the gut microbiota composition of MS patients who excluded independent influencing factors before and after WMT and donors. At the phylum level, the gut microbiota mainly included *Firmicutes, Fusobacteriota, Bacteroidota, Actinobacteriota*, and *Proteobacteria*. At the phylum level, the relative abundance of *Firmicutes, Bacteroidota*, and *Actinobacteriota* increased after WMT. The relative abundance of *Fusobacteriota* and *Proteobacteria* decreased (Figure 5A). At the family level, the relative abundance of *Bacteroideaceae, Lachnospiraceae, Bifidobacteriaceae*, and *Ruminococcaceae* increased, with a great increase of *Bifidobacteriaceae* in MS patients after WMT and donors, while the relative abundance of *Enterococcaceae* and *Fusobacteriaceae* decreased, with a great decrease of *Enterococcaceae* in MS patients after WMT and donors (Figure 5B). At the genus level, the relative abundance of *Bifidobacterium, Bacteroides, Faecalibacterium*, and *Prevotella* increased after WMT. The relative abundance of *Enterococcus, Fusobacterium, Escherichia–Shigella*, and *Klebsiella* reduced (Figure 5C).

The phylogenetic relationships of the top 100 gut microbiota at the genus level were analyzed, with the top seven being *Bacteroides, Fusobacterium, Bifidobacterium, Escherichia–Shigella, Faecalibacterium, Enterococcus*, and *Prevotella* (Figure 5D). Among them, not only could WMT increase the relative abundance of beneficial bacteria such as *Bifidobacterium* and *Bacteroidetes*, playing the basic functions of beneficial bacteria, but also reduce the relative abundance of pathogenic bacteria such as *Fusobacterium* and *Escherichia-Shigella*. WMT increased gut microbiota α diversity, such as Chao1 index (Figure 5E), revealing that WMT could significantly improve the diversity of gut microbiota in MS patients.

LEfSe analysis was performed on MS patients before and after WMT and donors to identify the biomarkers with statistical differences between groups. It was found that *Enterococcus* was the distinct species before WMT, while *UCG-002* was the distinct species after WMT in MS patients, and the distinct species was *Lactobacillaceae* in donors (Figure 5F). The species with significant differences between the MS patients before and after WMT were identified by Metastat. We found that it could still significantly increase the relative abundance of *Paraprevotella, Fournierella*, and *Senegalimassilia* compared to baseline WMT, with a great reduction of the relative abundance of *Hungatella* and *Ruminococcus* at the genus level (Figure 5G).

In conclusion, compared to baseline, the relative abundance of *Firmicutes, Bacteroidota*, and *Actinobacteriota* increased after WMT, while the relative abundance of *Fusobacteriota* and *Proteobacteria* decreased at the phylum level, maintaining the balance of gut microbiota (Figure 5H). In addition, the proportion of *Firmicutes/Bacteroidota* decreased after WMT and in the donor group. The relative abundance of gut microbiota in MS patients after WMT gradually tended toward healthy donors, suggesting that maintaining the homeostasis of gut microbiota might be a new approach for treating MS patients.

## 4 Discussion

As far as we know, our previous clinical study was the first to investigate the impact of WMT on MS patients in China, suggesting that the regulation of gut microbiota might be a new approach for treating MS (16). Therefore, WMT had been applied to treat numerous MS patients with gastrointestinal diseases. In this study, we found that WMT had a significant effect in removing the label of MS and downregulating ASCVD risk stratification. In addition, the therapeutic effect of WMT on MS patients was closely related to their age, smoking index, infection, chronic disease status, donor type and WMT courses based on Logistic Regression.

FMT is the process of transferring fecal bacteria extracted from healthy individuals to the patients' intestines through various optimization methods, thereby restoring intestinal microbiota homeostasis and providing effective treatment for a range of diseases both inside and outside the intestine. Scientific research has demonstrated the potential roles of gut microbiota in many diseases. In real-world clinical treatments, FMT has successfully treated various diseases, such as clostridium difficile infection (CDI) (27, 28) and inflammatory bowel disease (IBD) (29). However, FMT is not limited to digestive system diseases, it has also been proven to be a potential therapy for improving lipid (30, 31), blood glucose levels (32), MS (33, 34), obesity (35), sleep disorders (36) and hyperlipidemia (37) in recent years. Our early studies had shown that WMT significantly improves indicators such as FBG, TG, TC, LDL-c, SBP, and BMI in MS patients. In this study, the WMT group was more likely to remove the label of MS compared to the DT group based on improvements in aspects such as blood lipids, blood glucose and blood pressure.

Vrieze et al. (38) found that insulin sensitivity and the diversity of gut microbiota significantly increased in MS patients after transplanting healthy gut microbiota to for 6 weeks. The ratio of *Firmicutes* to *Bacteroidota* was commonly used to correlate changes in microbiota composition with obesity and type 2 diabetes mellitus (T2DM) phenotypes. Patients with obesity and T2DM had more *Firmicutes* than *Bacteroidota* in their intestines, while healthy people had fewer *Firmicutes* and more *Bacteroidota* (39, 40). Correspondingly, in this study, we not only observed an increase in gut microbial diversity and beneficial bacteria (such as Bifidobacterium and Bacteroidetes), but also a decrease in the proportion of Firmicutes/Bacteroidetes after WMT.

We believed that the improvement of gut microbiota might be key to treating MS patients with WMT. Therefore, the survival activity and diversity of gut microbiota after transplantation were crucial for the efficacy of WMT. Lange et al. found that the abuse of antibiotics could transform the gut microbiota into a long-term dysbiosis, potentially promoting the development and deterioration of diseases (41). de Oliveira et al. found that the diarrhea after 2019 corona virus disease (COVID-19) was related to the decrease of the abundance and diversity, as well as dysfunction of gut microbiota (42). In our study, the infection with pathogenic bacteria or viruses and the use of antibiotics significantly reduced the efficacy of WMT, suggesting minimizing pathogenic bacterial or viral infection and avoiding antibiotics after WMT. Zhang et al. (43) showed that the success of treating for ulcerative colitis (UC) with FMT was correlated with donor selection, believing that donor-recipient matching strategy was more effective in increasing the abundance of gut microbiota. Chehri et al. (44) showed that multiple courses could continuously improve the effectiveness of FMT in treating CDI. Similar to the study above, our study indicated that multiple donors and multiple courses could improve the efficacy of WMT, which suggested that the number of donors and frequency of WMT should be appropriately increased to achieve better efficacy.

The efficacy of WMT was also associated with patient age, lifestyle and dietary habits (45, 46). Coman and Vodnar (45) found that supplementing prebiotics or transplanting fecal microbiota could significantly improve the gut microbiota of elderly people and extend their health lifespan. In our study, individuals over 60 years old showed improved efficacy after WMT, likely due to significant gut microbiota improvements and high medical compliance. Moreover, a large number of studies had confirmed that smoking could cause oxidative stress, vascular inflammation, platelet coagulation, vascular dysfunction and dysregulate blood lipid levels, all of which could harm the cardiovascular system (47, 48). Consistent with this, our study supported that MS patients who are addicted to smoking had poorer efficacy, which was more difficult to remove the label of MS after WMT. As is well known, the homeostasis of the gut microbiota was closely related to good sleep time and exercise (49, 50). Therefore, we recommend that patients maintain regular schedules and exercise habits, while also advising them to quit smoking immediately.

To our knowledge, MS was a symptom of one or more high-risk factors such as centripetal obesity, hypertension, hyperglycemia and dyslipidemia (51, 52). Numerous studies had shown that patients with T2DM and obesity had metabolic disorders and chronic inflammatory states, accompanied by disturbances in gut microbiota (53, 54). On the contrary, if the gut microbiota was imbalanced for a long time, the abnormal situation of blood lipids, blood glucose and blood pressure would further worsen. Vich vila et al. believed that the use of hypoglycemic drugs such as Metformin could reduce the richness and diversity of gut microbiota (55). Xiong et al. showed that the pharmacokinetics and metabolism of antihypertensive drugs might be influenced by the gut microbiota (56). In our study, MS patients with one or more diseases such as hypertension, diabetes and obesity often took oral antidiabetic drugs or antihypertensive drugs for a long time, which made it more difficult to remove the label of MS. Therefore, we suggested that MS patients should strengthen monitoring of blood pressure, blood glucose and weight, as well as have a light diet, exercise reasonably, control weight and take medication reasonably.

It was known that many factors affect the efficacy of FMT, including the amount of transplanting feces, sample handling, injection method and colonization resistance (57, 58). Our WMT process was based on the standard of the Nanjing Consensus (20), which used automatic purification machines for repeated centrifugation and filtration to prepare washed microbial suspensions, thereby reducing differences in fecal volume and sample processing, as well as adverse reactions after transplantation. Due to the small sample size, further exploration was needed on the factors that affect the efficacy of WMT. However, the impact and influencing factors of gut microbiota on MS were still in its early stages, while the data on the impact and influencing factors of WMT on MS patients was limited. Our early research was the first large-scale retrospective study in China, indicating that WMT had a significant improvement effect on blood lipids, blood glucose, and blood pressure in MS patients (16). In this study, we further explored the factors influencing the efficacy of WMT in treating MS patients, laying the foundation for subsequent research on the effects of environmental factors (59), gut microbiota (60, 61) and metabolic biomarkers (62, 63) on metabolic diseases.

Our data had several limitations. Firstly, our study focused on single center data with a relatively low number of MS patients returning for evaluation of WMT efficacy. Therefore, more data was needed to confirm the factors influencing the efficacy of WMT in treating MS patients. Secondly, our study mainly focused on the analysis of clinical data and gut microbiota structure. A great deal of data such as the dietary structure, exercise frequency, sleep time, sample processing methods, and time of transplantation had not been evaluated yet. Therefore, more data should to be collected to further evaluate the factors that affect the efficacy of WMT. Thirdly, we did not evaluate the confusion factors between the main diseases for WMT treatment and MS. Although our research suggested that WMT had a significant effect in restoring MS to non-MS, and its therapeutic effect was closely related to patient age, smoking index, infection, chronic disease status, donor type and WMT course, the large scale prospective studies were needed to further approve our conclusions. In the future, we would continue to follow up all patients in this study and plan to conduct a large sample prospective study to verify the impact of WMT on MS patients and the influencing factors of WMT efficacy.

## 5 Conclusion

WMT has a significant effect in removing the label of MS and downregulating ASCVD risk stratification. The therapeutic effect of WMT on MS patients is closely related to their age, smoking index, infection, chronic disease status, donor type, and WMT courses. Therefore, we can improve the efficacy of WMT by reducing independent influencing factors that affect gut microbiota homeostasis.

## Data Availability

The original contributions presented in the study are included in the article/Supplementary material, further inquiries can be directed to the corresponding authors.
