# Supplementary material for: Analysis of influencing factors of washed microbiota transplantation in treating patients with metabolic syndrome
Source: Front Nutr. 2025 Feb 3;12:1508381. doi: 10.3389/fnut.2025.1508381 (PMC11830617; doi:10.3389/fnut.2025.1508381)
Supplement: Supplementary file 1 [file Data_Sheet_1.PDF]

Supplementary Table 1 The comparison values of each index inWMT group with baseline during the treatment of washed microbiota transplantation.

	**Baside1**	**After1**	***P1***	**Baside2**	**After2**	***P2***	**Baside3**	**After3**	***P3***
BMI	26.61±4.67(n=68)	26.05±4.61(n=68)	0.007	26.44±4.30(n=44)	25.74±4.28(n=44)	0.033	27.52±4.05(n=22)	27.04±4.16(n=22)	0.418
FBG (mmol/L)	6.36±2.37(n=63)	5.83±1.76(n=63)	0.038	6.15±2.39(n=43)	5.95±2.26(n=43)	0.409	6.34±2.69(n=20)	6.44±2.82(n=20)	0.785
FI (mU/mL)	14.17 (6.50-19.90) (n=23)	15.68 (7.75-19.78) (n=23)	0.543	10.72 (6.83-19.72) (n=23)	13.18 (6.91-23.03) (n=23)	0.418	14.39±6.78(n=7)	15.01±6.89(n=7)	0.787
HOMA-IR	3.40 (2.03-5.88) (n=23)	3.93 (2.03-6.36) (n=23)	0.927	3.40 (2.12-5.39) (n=13)	3.62 (1.95-7.89) (n=13)	0.566	4.00±2.01(n=7)	4.06±2.01(n=7)	0.931
TC (mmol/L)	5.07±1.84(n=63)	4.75±1.26(n=63)	0.143	5.03±1.60(n=42)	4.77±1.29(n=42)	0.278	5.15±1.46(n=21)	5.05±1.35(n=21)	0.624
TG (mmol/L)	3.20±4.04(n=63)	2.34±1.87(n=63)	0.013	2.43 (1.64-3.33) (n=42)	1.88 (1.34-3.19) (n=42)	0.068	3.41±2.65(n=21)	3.22±3.98(n=21)	0.786
LDL-c (mmol/L)	2.71±1.01(n=63)	2.67±1.03(n=63)	0.742	2.67±1.06(n=42)	2.65±1.19(n=42)	0.940	2.71±1.04(n=21)	2.64±0.79(n=21)	0.290
HDL-c (mmol/L)	0.99±0.25(n=63)	1.10±0.45(n=63)	0.029	0.94 (0.78-1.17) (n=42)	1.01 (0.90-1.16) (n=42)	0.086	0.98±0.25(n=21)	1.01±0.23(n=21)	0.280
SBP (mmHg)	133.12±12.01(n=68)	125.78±12.56(n=68)	＜0.001	132.49±11.82(n=45)	125.64±11.64(n=45)	0.006	129.82±12.25(n=22)	118.68±10.51(n=22)	0.010
DBP (mmHg)	82.78±9.69(n=68)	77.81±9.40(n=68)	0.002	82.56±9.65(n=45)	79.18±8.23(n=45)	0.037	82.05±9.96(n=22)	75.18±10.05(n=22)	0.024

Data is represented as mean ± standard deviation or interquartile spacing.

The HbA1c data is too limited to be statistically analyzed. After 1 refers to the indicators after the first WMT treatment, and so on.

Supplementary Table 2 Comparison of improvement values for various indicators during the single-course of treatment in the WMT group.

	**WMT-ND group (n=43)**	**WMT-D group (n=25)**	**P**
△BMI (kg/m2)	0.77±1.92(n=43)	0.20±0.92(n=25)	0.174
△FBG (mmol/L)	0.37±1.46(n=38)	0.77±2.61(n=25)	0.443
△HbA1c (%)	0.20±0.53(n=3)	0.16±0.51(n=10)	0.908
△FI (mU/mL)	2.08±9.41(n=13)	-2.35±4.23(n=10)	0.183
△HOMA-IR	1.05±3.30(n=13)	-0.53±2.17(n=10)	0.205
△TC (mmol/L)	0.18±1.23(n=45)	0.67±2.58(n=18)	0.316
△TG (mmol/L)	0.62±1.76(n=45)	1.81±4.81(n=18)	0.153
△LDL-c (mmol/L)	0.04±0.97(n=45)	0.05±1.15(n=18)	0.965
△HDL-c (mmol/L)	-0.08±0.24(n=45)	-0.18±0.62(n=18)	0.353
△SBP (mmHg)	7.38±14.40(n=34)	7.29±17.41(n=34)	0.982
△DBP (mmHg)	6.26±9.88(n=34)	3.68±14.74(n=34)	0.398

Data is represented as mean ± standard deviation.

△BMI refers to the improvement value in BMI, and so on. WMT-ND group represents WMT without drug treatment and WMT-D group represents WMT plus drug treatment.

Supplementary Table 3 Comparison of improvement values for various indicators between the DT group and WMT group.

	**WMT group (n=68)**	**DT group (n=142)**	**P**
△BMI (kg/m2)	0.56±1.64(n=68)	0.12±1.33(n=122)	0.047
△FBG (mmol/L)	0.53±1.99(n=63)	-0.41±2.57(n=123)	0.012
△HbA1c (%)	0.17±0.49(n=13)	0.15±1.38(n=109)	0.965
△FI (mU/mL)	0.15±7.78(n=23)	1.11±6.22(n=4)	0.819
△HOMA-IR	0.36±2.92(n=23)	0.13±1.45(n=4)	0.877
△TC (mmol/L)	0.32±1.72(n=63)	0.25±1.01(n=133)	0.732
△TG (mmol/L)	0.96±2.97(n=63)	0.02±1.67(n=133)	0.005
△LDL-c (mmol/L)	0.04±1.01(n=63)	0.23±0.95(n=133)	0.204
△HDL-c (mmol/L)	-0.11±0.25(n=63)	-0.04±0.24(n=133)	0.098
△SBP (mmHg)	7.34±15.86(n=68)	1.97±18.00(n=142)	0.037
△DBP (mmHg)	4.97±12.52(n=68)	0.99±13.74(n=142)	0.045

Data is represented as mean ± standard deviation.
